# Micro-cold-forming: a simple, rapid, and inexpensive method for the fabrication of microcavities for 3D cell culture

**DOI:** 10.1039/d6ra02741e

**Published:** 2026-05-15

**Authors:** Jay Rabindra Kumar Samal, Pinak Samal, Carla Pou Casellas, Maarten B. Rookmaaker, Marianne C. Verhaar, Pamela Habibović, Stefan Giselbrecht, Roman Kurt Truckenmüller

**Affiliations:** a MERLN Institute for Technology-Inspired Regenerative Medicine, Maastricht University 6229 ER Maastricht The Netherlands j.samal@maastrichtuniversity.nl r.truckenmuller@maastrichtuniversity.nl; b Department of Nephrology and Hypertension, University Medical Center Utrecht 3584 CX Utrecht The Netherlands; c Hubrecht Institute for Developmental Biology and Stem Cell Research – Royal Netherlands Academy of Arts and Sciences 3584 CT Utrecht The Netherlands

## Abstract

Recently, microcavity arrays have been increasingly used to enable the controlled culture of 3D cellular aggregates, such as spheroids, organoids, and gastruloids. However, current fabrication techniques remain technically demanding and largely inaccessible. While micro(scale) thermoforming is already used for fabricating thin-walled microcavities in films from thermoplastic polymers, the process requires specialized equipment for the controlled application of forming temperature and pressure. Here, a new microfabrication method, referred to by us as “micro-cold-forming”, is described. This method enables the simple, rapid, and inexpensive fabrication of microcavities (with diameters of 2 mm and depths ranging from 530 to 690 µm) in thin (25–30 µm) thermoplastic polymer films. The fabrication method is carried out at ambient or room temperature and only uses commercially available, easily affordable components. Here, we demonstrate that the microcavities can be applied for 3D cell culture by culturing adult human kidney organoids, called “tubuloids”, in the microcavities. To demonstrate the ability of the microcavities to be applied for toxicity studies, the tubuloids were exposed to a high concentration of ascorbic acid. The new forming technique can provide widespread, low-barrier access to self-fabricated microcavities for researchers both in biological laboratories and in low-income countries.

## Abstract 2

An ambient-temperature micro-cold-forming technique enables straightforward, rapid, and low-cost fabrication of polymer microcavities for 3D organoid culture and toxicity testing.

## Graphical abstract

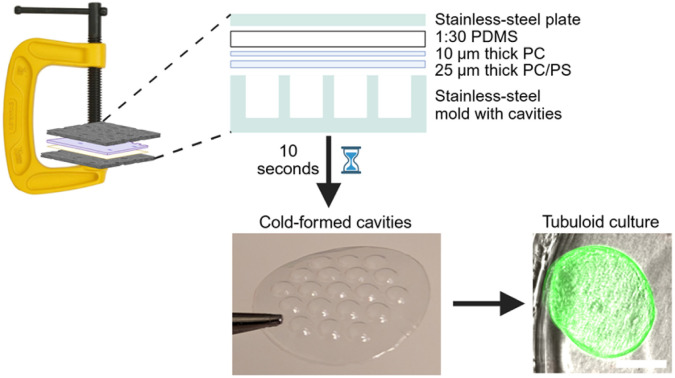

## 1 Introduction

Microcavities have been increasingly applied over the past few years for the culture of intestinal organoids,^1–3^ bronchial organoids,^4^ and gastruloids,^5,6^ to name a few. Regularly arranged in arrays, microcavities can enable the culture of organoids at defined *x*-, *y*-, and *z*-positions. These allow for high-throughput manipulation and imaging of organoids, with unobstructed access to each individual organoid – advantages not possible in conventionally applied drops of basement membrane extract (BME), where organoids are also less uniform in size, shape, and inter-organoid spacing.^7,8^ Thin-walled microcavities for 3D cell culture have already been fabricated in thin thermoplastic polymer films using micro thermoforming.^9–13^ The polymers polycarbonate (PC) and polystyrene (PS) can both be processed by micro thermoforming; they are bioinert and do not absorb small molecules in the same way polydimethylsiloxane (PDMS) does, making them ideal for toxicity and drug efficacy testing.^14^ However, the micro-thermoforming process is not trivial due to the required controlled and coordinated application of (high) temperatures and pressures, which in turn requires specialized equipment. Most polymers require forming temperatures above 100 °C,^15^ which results in extended cooling times for the thermoforming mold and, consequently, the formed film.^16,17^ If water is used as a coolant, the steam thereby generated often necessitates an extra venting step. Additionally, for micro pressure (thermo)forming, higher gas pressures can be challenging, as they pose safety risks and require specialized equipment that can safely handle them. These challenges have rendered the technique largely inaccessible to many researchers, particularly those without access to facilities with specialized equipment, such as researchers in biological laboratories and in low-income countries.

Here, we describe a novel microfabrication technique, which we refer to as “micro-cold-forming”, for the simple, rapid, and inexpensive fabrication of microcavities in thermoplastic polymer films. The fabrication process can be carried out at ambient or room temperature (RT), eliminating the need for heating and cooling equipment and the associated thermal cycle time. Furthermore, pressure is applied to the film to be formed using readily available and affordable clamps and a PDMS slab, which is considerably safer than applying gas pressure and requires no specialized equipment. The process was designed to reproducibly obtain microcavities in films made from the well-established culture substrate polymers PC and PS. To demonstrate the applicability of the fabricated microcavities for 3D culture, we cultured adult human kidney organoids, so-called “tubuloids”,^18^ in the platform under suspension culture conditions. As kidneys are one of the primary targets for drug-induced nephrotoxicity, developing *in vitro* cell culture platforms for toxicity screening is critical for preclinical safety assessments.^19^ To achieve this, we demonstrated the applicability of the microcavities for toxicity studies by exposing the tubuloids to a high concentration of ascorbic acid (vitamin C). We found that tubuloids cultured in the microcavities successfully maintained high viability, apico-basal polarity, and kidney-specific marker expression comparable to conventional BME dome-embedded cultures. Furthermore, the tubuloids demonstrated marked cell death upon exposure to ascorbic acid compared with control tubuloids, validating the platform's applicability for high-content toxicity screening.

## 2 Materials and methods

### 2.1 Materials and reagents

PC films (10 µm and 25 µm thick) were obtained from it4ip. PS films (30 µm thick) were obtained from Goodfellow. The SYLGARD 184 elastomer kit (PDMS) was obtained from Dow. BIOFLOAT FLEX was obtained from faCellitate and was used to prevent cell attachment. Tubuloids were sourced from the Hubrecht Organoid Technology (HUB). Cultrex Reduced Growth Factor Basement Membrane Extract was obtained from R&D Systems. Advanced DMEM/F-12, GlutaMAX, B27 supplement, penicillin-streptomycin, BlockAid Blocking Solution, rabbit anti-ZO-1, Alexa Fluor 647 Phalloidin, Hoechst 33342, Alexa Fluor 647-conjugated donkey anti-rabbit IgG, and Alexa Fluor 568-conjugated donkey anti-mouse IgG were obtained from Thermo Fisher Scientific (Gibco). Rspo1-conditioned medium was obtained from U-Protein Express. EGF and FGF-10 were obtained from PeproTech. A 83-01 was obtained from Tocris Bioscience. Rabbit anti-AQP-3 and mouse anti-Integrin ɑ6 were obtained from Abcam while mouse anti-acetylated tubulin was obtained from Santa Cruz. For live/dead staining, dyes calcein AM and ethidium homodimer-1 were obtained from Invitrogen. Paraformaldehyde and Triton X-100 were obtained from VWR. HEPES, *N*-acetylcysteine, and ascorbic acid were obtained from Sigma-Aldrich. RNeasy Micro Kit was obtained from Qiagen and iScript cDNA Synthesis Kit and iQ SYBR Green Supermix were obtained from Bio-Rad.

### 2.2 Cold forming of microcavity arrays

**Fig. 1:**
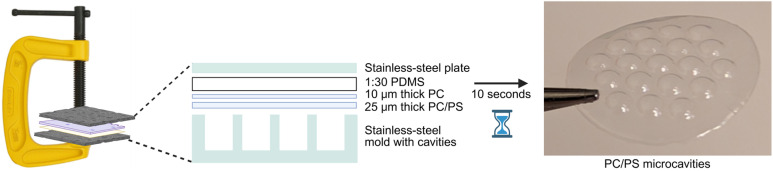
Schematic of micro-cold-forming process for fabrication of microcavities. A stack consisting of an approximately 25 µm thick PC or 30 µm thick PS film, a 10 µm thick PC film, a 1 : 30 PDMS slab, and a stainless-steel plate were placed on top of the mold and pressure was applied with a dwell time of 10 s to fabricate the microcavities. The 19 hexagonally arranged circular microcavities of the array have on their backside an outer diameter of 2 mm and a minimum inter-cavity spacing of 0.25 mm.

For forming the microcavities, a mold with 19 hexagonally arranged cylindrical holes, each with a diameter of 2 mm, a depth of 2 mm, and a minimum spacing of 0.25 mm between them, was designed using Autodesk Inventor 2018 (Autodesk) and precision-milled into a 4 mm thick SAE 316 stainless-steel plate (Xometry) (Fig. S1). A 1 mm thick counter plate, but without microcavities, was also fabricated. After fabrication, the mold plate and the counter plate were first cleaned with acetone, followed by ethanol, and then dried. For cold forming PC microcavities, a stack was placed on top of the mold consisting of, first, the nominally 25 µm thick PC film (to be formed) and an additional, 10 µm thick PC film (both it4ip), then a 2.845 ± 0.062 mm thick cured PDMS (Dow, SYLGARD 184 elastomer kit; 1 : 30 ratio of curing agent to PDMS prepolymer; hereafter referred to as “1 : 30 PDMS”) slab, and finally a 1 mm thick SAE 316 stainless-steel plate. The additional PC film was introduced to prevent the PDMS slab from sticking to the formed film after forming. To fabricate the microcavities, the stack was clamped using a steel C-clamp (Stanley Black & Decker Deutschland) capable of applying a clamping force equivalent to 680 kg. The screw of the clamp was maximally hand-tightened to apply pressure with a dwell time of 10 s before reducing the pressure (Fig. 1). For fabricating PS microcavities, the same process and setup were employed, but using a nominally 30 µm thick PS film (Goodfellow, Cat. no. 82789543) instead of the 25 µm thick PC film. PC and PS were deliberately selected because they are bioinert materials commonly used in commercial tissue culture plasticware. These materials do not absorb small molecules and offer high optical transparency for downstream imaging.

### 2.3 Thickness measurement of PC and PS films, scanning electron microscopy inspection and surface roughness and depth measurement of microcavity arrays

The thickness of the PC and PS film was measured using a length gauge (Heidenhain, MT 60M). Visual inspection of microcavity arrays was performed using a scanning electron microscope (Thermo Fisher Scientific/FEI, Teneo). For this, the arrays were mounted on aluminum stubs using conductive carbon adhesive tabs and sputter-coated with iridium. Surface roughness (areal roughness, Sa) and microcavity depth were measured using a 3D confocal laser scanning microscope-based optical profilometer (Keyence, VK-X250).^20^

### 2.4 Autofluorescence measurements

Autofluorescence of flat and formed PC and PS films was measured using a fluorescence microscope (Nikon, Eclipse Ti-S). The measurements were conducted at 390, 488, 568, and 647 nm, with the same settings across all samples.

### 2.5 Preparation of microcavity arrays for cell culture

After cold forming, the formed microcavity arrays were punched using a manual punch and mounted into 24-well plates with polymer coverslip bottom (ibidi, µ-Plate 24 Well, Cat. no. 82421) using ethylene propylene diene monomer (EPDM) O-rings (ERIKS, Cat. no. 559273). Prior to cell culture, the microcavity arrays were sterilized by dipping in 70% ethanol for 15 min, followed by two washes with sterile Dulbecco's phosphate buffered saline (PBS; Sigma-Aldrich). In order to prevent cell attachment, the arrays were provided with a low-binding coating by incubating them in a corresponding solution (faCellitate, BIOFLOAT FLEX) as per the manufacturer's instructions.

### 2.6 Tubuloid culture

The tubuloids (Hubrecht Organoid Technology, HUB code HUB-05-A2-003) were cultured as previously described.^18^ Briefly, fragments were seeded in domes of Cultrex Reduced Growth Factor Basement Membrane Extract (BME; R&D Systems) in Advanced DMEM/F-12 medium (Gibco) supplemented with 1% penicillin-streptomycin (Thermo Fisher Scientific), 1% HEPES (Sigma-Aldrich), 1% GlutaMAX (Gibco), 1.5% B27 supplement (Gibco), 10% Rspo1-conditioned medium (U-Protein Express), 50 ng ml^−1^ EGF (PeproTech), 100 ng ml^−1^ FGF-10 (PeproTech), 1.25 mM *N*-acetylcysteine (Sigma-Aldrich), and 5 µM A 83-01 (Tocris Bioscience), and cultured at 37 °C and 5% CO_2_. Medium changes were performed every 2 days, and the tubuloids were passaged every 7 days. For suspension culture of the tubuloids in the cold-formed PC microcavities, tubuloid fragments were resuspended in the supplemented Advanced DMEM/F-12 medium containing 10% v/v BME and seeded into the microcavities.

### 2.7 Live/dead assay

Tubuloid viability was assessed by simultaneously staining viable and dead cells with the dyes calcein AM (Molecular Probes, Invitrogen) and ethidium homodimer-1 (EthD-1; Molecular Probes, Invitrogen), respectively, according to the manufacturer's instructions. The tubuloids were imaged in the microcavities and BME domes using a fluorescence microscope (Nikon ECLIPSE Ti-S). Quantification of viability was performed by determining the percentage of area positively stained for EthD-1 using ImageJ.

### 2.8 Immunostaining

Tubuloids from BME domes were extracted using cold Gentle Cell Dissociation Reagent (STEMCELL Technologies). After centrifugation at 100×*g* for 5 min at 4 °C, the supernatant was removed and the tubuloids were fixed with 4% paraformaldehyde (VWR) in PBS for 30 min. For the (PC) microcavities, the tubuloids were directly fixed on-chip with 4% paraformaldehyde in PBS for 30 min. Subsequently, the tubuloids were permeabilized with 0.5% Triton X-100 (VWR) in BlockAid Blocking Solution (Thermo Fisher Scientific) at RT and washed twice with PBS. Then, the tubuloids were incubated with primary antibodies in BlockAid Blocking Solution with 0.3% Triton X-100 overnight at 4 °C and washed twice with PBS. Finally, the secondary antibodies and the F-actin and nuclear staining solutions were added, and the tubuloids were incubated for 3 h at RT, and washed twice with PBS.

The primary antibodies included rabbit anti-AQP-3 (1 : 100; Abcam, Cat. no. ab125219), mouse anti-acetylated tubulin (1 : 2000; Santa Cruz, Cat. no. sc-23950), rabbit anti-ZO-1 (1 : 100; Thermo Fisher Scientific, Cat. no. 40-2200), and mouse anti-Integrin ɑ6 (1 : 200; Abcam, Cat. no. ab20142). Secondary antibodies included Alexa Fluor 647-conjugated donkey anti-rabbit immunoglobulin G (IgG) (1 : 250; Thermo Fisher Scientific) and Alexa Fluor 568-conjugated donkey anti-mouse IgG (1 : 250; Thermo Fisher Scientific). Apart from the immunostaining, as indicated, F-actin and nuclei were stained, which was performed using Alexa Fluor 647 Phalloidin (1 : 500; Thermo Fisher Scientific, Cat. no. A22287) and Hoechst 33342 (1 : 1000; Thermo Fisher Scientific, Cat. no. H3570), respectively.

### 2.9 Confocal fluorescence imaging

The fluorescently stained aggregates were imaged using a spinning-disk confocal fluorescence microscope (Nikon, ECLIPSE Ti-S) with 60× oil immersion and 20× low-working distance objectives. For imaging with the higher magnification objective, the tubuloids were placed in 35 mm Petri dishes with glass cover slip bottom (ibidi, Glass Bottom Dish 35 mm, Cat. no. 81218-200) in Dako fluorescence mounting medium (Agilent). Images were processed and analyzed using NIS-Elements (Nikon) or FIJI (https://fiji.sc/) and subsequently assembled into figures using the QuickFigures plugin for FIJI.^21^

### 2.10 Quantitative polymerase chain reaction analysis

Tubuloids from cold-formed PC microcavities or BME domes were dissociated using cold Gentle Cell Dissociation Reagent (GCDR; STEMCELL Technologies), followed by centrifugation at 300×*g* for 5 min at 4 °C. Total RNA was extracted using RNeasy Micro Kit (Qiagen) as per the manufacturer's instructions, followed by cDNA synthesis using iScript cDNA Synthesis Kit (Bio-Rad). Quantification of gene expression was carried out using iQ SYBR Green Supermix (Bio-Rad) for quantitative (real-time) polymerase chain reaction (qPCR) in a PCR machine (Bio-Rad, CFX96 Real-Time PCR Detection System) applying the following protocol: denaturation at 94 °C for 5 min, followed by 60 cycles at 94 °C for 15 s, 60 °C for 20 s, and 72 °C for 40 s, and melt curve analysis at 55–90 °C with a 0.5 °C increase every 3 s. Gene expression was normalized using glyceraldehyde-3-phosphate dehydrogenase (GAPDH) as a housekeeping gene. Data were analyzed using the 2^−ΔΔ*C*_t_^ method. Primers used for gene expression analysis are listed in the supplementary information (SI) of this manuscript (Table S1).

### 2.11 Ascorbic acid exposure

For the ascorbic acid toxicity study, 30 mM ascorbic acid was added to the medium after 3 days of tubuloid culture in cold-formed PC microcavities. The tubuloids were incubated in this medium for 24 h at 37 °C and 5% CO_2_. Tubuloid viability was assessed using live/dead staining. Tubuloids cultured with medium without additional ascorbic acid for the same period of time served as control.

### 2.12 Statistical analysis

In the bar graphs, the bars represent mean values, and the error bars represent standard deviations (SD). Depending on the data set, statistical significance was determined by either an unpaired *t*-test with Welch's correction for comparisons between two groups or two-way ANOVA followed by Bonferroni's post hoc multiple comparison test, using GraphPad Prism (GraphPad Software). For all samples, at least three measurements were considered (*N* ≥ 3). *P* < 0.05 was considered statistically significant for all tests.

## 3 Results and discussion

### 3.1 Cold forming and characterization of microcavity arrays

**Fig. 2:**
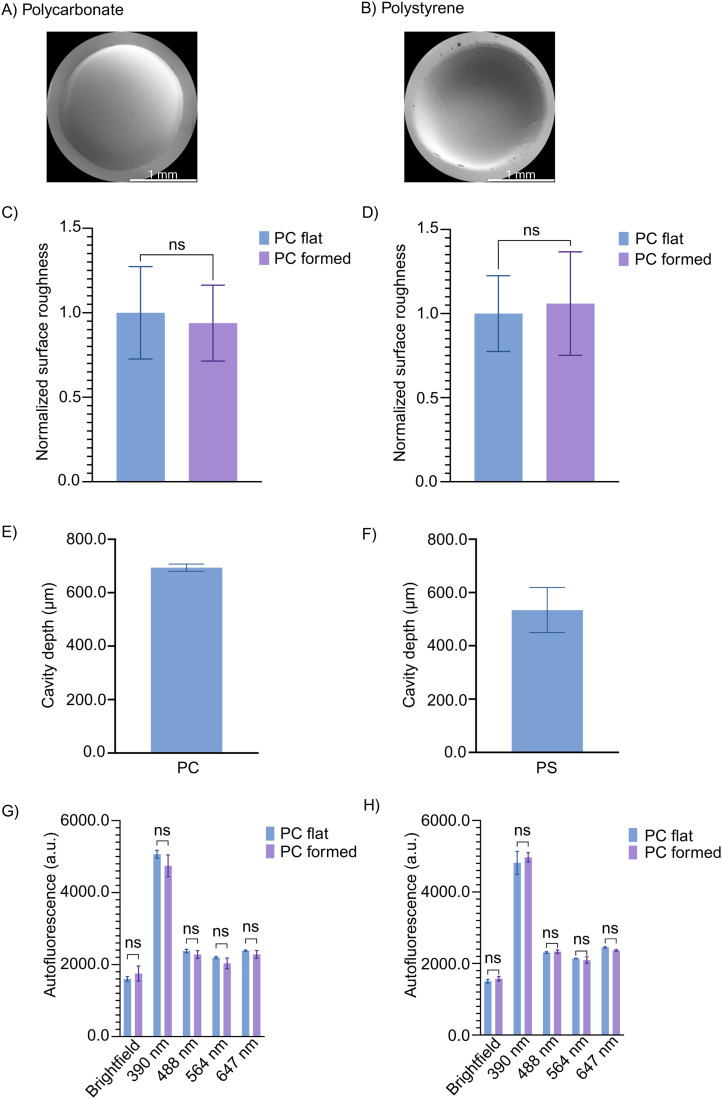
Characterization of cold-formed microcavities. SEM images of (A) PC and (B) PS microcavities (backside views). Surface roughness of micro-cold-formed and non-processed flat (C) PC and (D) PS films. All values were normalized to the corresponding flat control films. Data were analyzed using an unpaired *t*-test with Welch's correction. *N* = 3. Depth of (E) PC and (F) PS cavities measured using an optical profilometer. Bars and error bars represent mean values ± SD. Autofluorescence measurements of micro-cold-formed and non-processed (flat) (G) PC and (H) PS films. Bars and error bars represent mean values ± SD. Data were analyzed using two-way ANOVA followed by Bonferroni's post-hoc multiple comparison test. *N* = 3. a.u. and ns refer to arbitrary units and not significant, respectively.

The measured thicknesses of the PC and PS film were 25.8 ± 1.2 µm and 32.3 ± 0.27 µm, respectively (Fig. S2A and B, respectively). The fabricated microcavities from both PC and PS exhibited smooth surfaces, and no irregularities from an approximately spherical shape could be observed by scanning electron microscopy (SEM) (Fig. 2A and B). No significant increase in surface roughness was observed on micro-cold-formed films compared to non-processed flat control films (Fig. 2C and D). This is important as microscale topographies or roughness can markedly influence cell adhesion kinetics^22,23^ and reduce optical transparency during imaging. The depths of the PC and PS microcavities were measured using optical profilometry and found to be 694.1 ± 13.5 µm and 534.4 ± 84.5 µm, respectively (Fig. 2E and F). Even with hand-tightening of the C-clamp, the forming depth for the PC microcavities was highly reproducible. A lower depth and greater variability in this parameter were observed in PS microcavities compared to PC. This may be attributed to differences in the mechanical properties between the two polymers.^24^ At RT, PC exhibits more pronounced viscoplastic deformation and creep behavior than PS. Additionally, the PS film is biaxially oriented. The PS film is also slightly thicker, by approximately 25%. For oriented and/or thicker films, the equilibrium between the forces exerted by the deformed PDMS slab and the stretched film during forming is reached at a shallower forming depth than for non-oriented and/or thinner films of the same material. Based on the above results, all experiments reported in section 3.2 and subsequent sections were conducted using the PC film.

Apart from the film material (PC compared to PS), we also investigated the influence of the PDMS slab thickness and the thickness of the (PC) film (to be formed) on the forming process and the resulting forming depth. Increasing the thickness of the PDMS slab beyond the previously mentioned approximately 2.8 mm did not lead to a significant increase in microcavity depth. With regard to film thickness, thinner PC films of 10 µm could be successfully formed but lacked sufficient rigidity for robust handling and often collapsed or wrinkled after micro-cold-forming. In contrast, thicker films of 50 µm exhibited significantly reduced cavity depths for both PC and PS. Additionally, attempts to apply the micro-cold-forming process to ion track-etched porous PC films resulted in extensive crack formation. The pores may serve as sites for crack initiation. One potential solution could be cold forming heavy-ion-irradiated but not yet etched PC films and etching the pores only after forming the film, as we have previously demonstrated in combination with micro thermoforming.^9,25^ In summary, polymer type and initial film thickness influence the final structural integrity and depth of the cold-formed cavities. For the selected design parameters, optimal results were observed for intermediate thicknesses (25–30 µm), as thinner films lacked mechanical stability after the cold-forming process and thicker films exhibited limited formability at ambient temperature.

As fluorescence microscopy is a widely used technique for cell assays, the autofluorescence of microcavities in PC and PS across the four most commonly used wavelengths – 390, 488, 568, and 647 nm – was measured.^6,26^ No significant increase in autofluorescence was observed at any wavelength compared to the corresponding control films (Fig. 2G and H). Thus, the micro-cold-forming process is not expected to interfere with imaging experiments.

Microcavities for 3D cell culture can be fabricated by a wide range of processes, either directly – for example, by microdrilling or laser micromachining – or by polymer micromolding. The latter includes replica molding of materials such as PDMS or agarose hydrogels over a lithographic master, hot embossing, and microthermoforming. Compared to the most closely related process, microthermoforming, micro-cold-forming offers both advantages and disadvantages. Cold forming is carried out at ambient temperature, which drastically reduces equipment costs. Microthermoforming employs temperature-controlled presses that typically cost thousands of euros, while micro-cold-forming requires only simple hand clamps costing less than 10 euros. Because the heating and cooling times, which are 10 minutes or more, are eliminated, cycle times are reduced to just a few seconds. However, the easy micro-cold-forming process, in its current form as described here, is limited to a forming depth-to-feature width aspect ratio of the spherical microcavities of around 0.3, in contrast to ratios above 1 for microthermoforming.

### 3.2 Tubuloid viability in the cold-formed microcavities

**Fig. 3:**
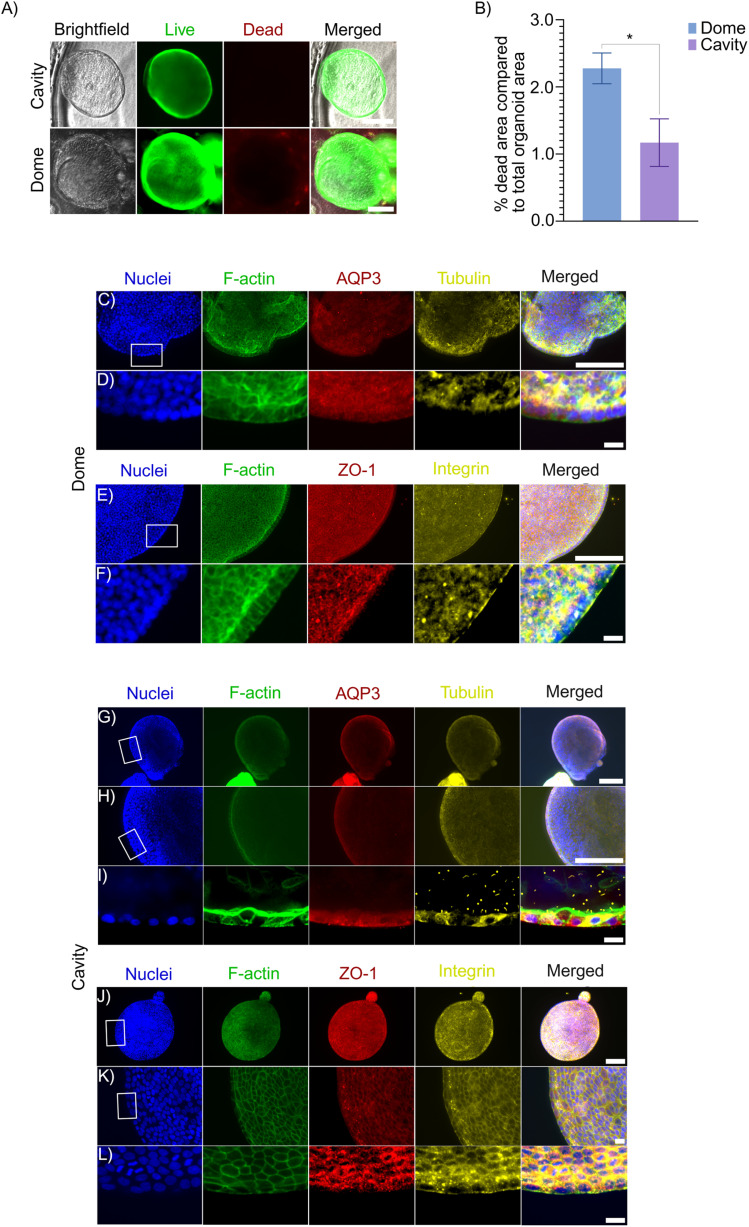
Tubuloid culture in microcavities. (A) Live/dead staining of tubuloids cultured in microcavities and BME domes after 4 days in culture. Scale bars represent 200 µm and apply to all images in the same row. (B) Quantification of percentage dead area compared to total tubuloid area. Bars and error bars represent mean values ± SD. Data were analyzed using an unpaired *t*-test with Welch's correction. *p < 0.05. *N* = 3. (C–L) Immunostaining of tubuloids cultured in microcavities and BME domes after 4 days in culture. Scale bars represent (C, E, G, H, and J) 200 µm, and (D, F, I, K, and L) 20 µm, and in each case apply to all images in the same row. AQP3, Tubulin, ZO-1, and Integrin refer to Aquaporin-3, acetylated tubulin, zonula occludens-1, and Integrin ɑ6, respectively.

To demonstrate the applicability of the fabricated microcavities for 3D culture, we cultured tubuloids in the microcavities under suspension conditions with 10% BME added to the medium. Live/dead staining of the tubuloids cultured in the microcavities and in the BME domes after 4 days in culture was performed using calcein AM/EthD-1 (Fig. 3A). Quantification of the percentage of dead area within the tubuloids revealed a higher dead area in tubuloids cultured in domes compared to those cultured in microcavities (Fig. 3B). The increased cell death in the domes is likely due to restricted diffusion of oxygen, nutrients, and metabolic waste products compared to the microcavities.^2,27,28^ In conventional BME domes, the dense hydrogel matrix acts as a barrier that limits mass transport. In contrast, in the microcavities, the tubuloids are cultured in suspension, thereby facilitating efficient exchange of soluble factors with the surrounding bulk medium.

### 3.3 Expression of apico-basal polarity and kidney-specific markers in the cold-formed microcavities

After 4 days in culture, tubuloids grown in the microcavities resembled tubuloids cultured in BME domes (Fig. 3A and C–L). At this time point, the tubuloids were stained to assess apico-basal polarity and kidney-specific markers (Fig. 3C–L). The expression patterns and localization of all assessed markers were similar between tubuloids cultured in microcavities and those cultured in BME domes. This suggests that suspension culture in micro-cold-formed cavities can support the maintenance of the tubuloid phenotype. Apico-basal polarity was evaluated using acetylated tubulin (cilia, apical surface), ZO-1 (tight junctions, apico-lateral region), and AQP3 (basolateral membrane). The establishment of this apico-basal polarity was comparable between the two conditions. In the kidney, the proper polarization of these proteins is essential for renal function, as it enables the transport of water and solutes across the tubular epithelium.^29^ The presence of primary cilia (acetylated tubulin) together with the localization of ZO-1 to the apico-lateral junctions in tubuloids cultured in both domes and cavities indicated the formation of a functional barrier separating the luminal and interstitial compartments. Kidney-specific markers included AQP3 (basolateral membrane of collecting duct principal cells; also present in other epithelia but enriched in kidney) and integrin α3 (basal membrane of tubular epithelial cells). The expression of these identity markers was preserved in microcavity-cultured tubuloids and was comparable to that in control tubuloids in BME domes. Co-expression of AQP3 and acetylated tubulin confirmed the presence of ciliated collecting duct cells (principal cells).

**Fig. 4:**
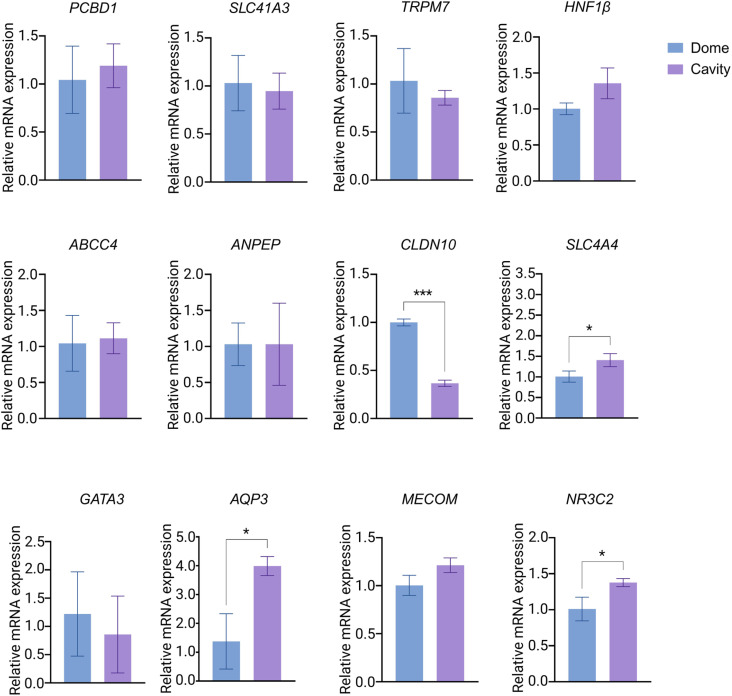
Gene expression analysis of tubuloids grown in domes and microcavities on day 4 of culture. Bars and error bars represent mean values ± SD. Data were analyzed using an unpaired *t*-test with Welch's correction. *p < 0.05 and ***p < 0.001. *N* = 3.

To assess the expression of markers representing different kidney regions, we performed qPCR for tubuloids cultured in microcavities and BME domes after 4 days of culture (Fig. 4). The analyzed genes and their associated nephron segments and functions are listed in the SI of this manuscript (Table S2).^30^ Distinct differences in gene expression were noted for specific transport markers. *SLC4A4* (proximal tubule), *NR3C2* (mineralocorticoid receptor), and *AQP3* (collecting duct) were significantly upregulated in tubuloids from microcavities compared to those from BME domes, suggesting improved functional maturation in the microcavity environment. This may be due to more efficient mass transport in the suspension culture within the microcavities, which likely mitigates the metabolic stress compared to conventional gel-embedded cultures with restricted diffusion. Such mitigation of metabolic stress may sustain the energy-intensive process of transporter synthesis, leading to the higher gene expression observed. Additionally, *CLDN10* gene expression was significantly downregulated in tubuloids from microcavities compared to BME domes. Previous studies have suggested that expression of tight junction proteins is influenced by mechanical forces from the surrounding environment.^31^ The plausibly reduced mechanical forces exerted on the tubuloids in suspension culture, compared to BME domes, may have contributed to the downregulation of *CLDN10*. Future studies are warranted to elucidate mechanotransduction pathways driving gene expression differences between suspension- and BME dome-cultured organoids.

### 3.4 Ascorbic acid toxicity study

**Fig. 5:**
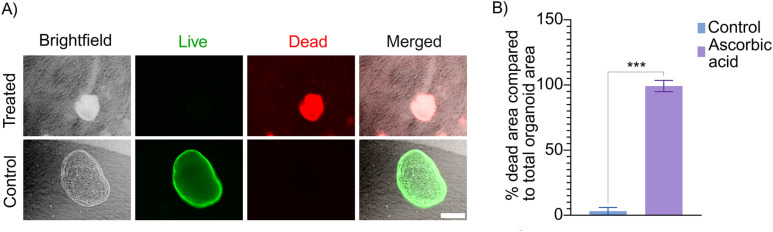
Ascorbic acid toxicity study. (A) Live/dead staining of tubuloids exposed to 30 mM ascorbic acid and control tubuloids. Scale bar represents 200 µm and applies to all images. (B) Quantification of the percentage of dead area compared to the total tubuloid area. Bars and error bars represent mean values ± SD. Data were analyzed using an unpaired *t*-test with Welch's correction. ***p < 0.001. *N* = 3.

To demonstrate the applicability of the platform for toxicity studies, tubuloids cultured in cold-formed microcavities were exposed to 30 mM ascorbic acid for 24 hours, followed by live/dead staining to assess viability. While ascorbic acid functions as an antioxidant at physiological levels, higher concentrations (mM range) can exert severe cytotoxic effects.^32,33^ Additionally, excessive intake of ascorbic acid has been linked to oxalate nephropathy and acute kidney injury (AKI) in patients.^34^ Live/dead staining revealed marked cell death in tubuloids exposed to ascorbic acid compared to control tubuloids. The (significant) effect was confirmed by quantification of the percentage of dead area in the tubuloids (Fig. 5). This suggests that micro-cold-formed cavities can be a viable tool for drug and toxicity screening.

## 4 Conclusions and outlook

We developed a simple, rapid, and inexpensive micro-cold-forming technique for the fabrication of microcavities in thermoplastic polymer films without the requirement of specialized equipment or even cleanrooms. We then demonstrated the applicability of the fabricated microcavities for 3D cell culture by optimizing the suspension culture of tubuloids. We compared the viability and the polarization and kidney marker expression to tubuloids cultured in BME domes. Next, we performed a toxicity study with ascorbic acid to demonstrate the applicability of the microcavities for toxicity screening. Due to its straightforward and cost-effective nature, micro-cold-forming is readily accessible both in biological laboratories and in low-income countries.

As the primary aim of this study was to develop an accessible, low-barrier platform tailored for 3D cell culture, we focused our systematic investigations on process parameters directly relevant to this biological application, including variations in film materials (PC and PS), film thicknesses, and PDMS properties. For the mold, we specifically utilized a simple, millimeter-sized circular design. This geometry was chosen because circular features can be easily fabricated (*e.g.*, by drilling), aligning with our intention to develop a process suitable for low-income settings. Furthermore, the resulting U-shaped cavity bottom is ideal for cell culture, as it promotes gravity-driven cell sedimentation to the deepest point to facilitate 3D aggregate formation. A millimeter-sized cavity is also highly versatile, capable of accommodating a wide spectrum of sizes ranging from small spheroids to millimeter-sized organoids. However, for applications requiring alternative base geometries (*e.g.*, elongated structures) or more sharply defined profiles rather than smoothly rounded bottoms, future studies will be required to systematically define the geometrical limits and replication fidelity of the micro-cold-forming process.

Future studies should investigate alternative thermoplastic films for cold-formed microcavities in 3D cell culture. Candidate materials that are simultaneously cold-formable, available as thin films (some are already biaxially oriented, though), bio- or cytocompatible, highly transparent, and resistant to chemicals commonly used in cell culture workflows include polyethylene terephthalate (PET), polyethylene terephthalate glycol (PETG), cyclic olefin polymers (COP), and cyclic olefin copolymers (COC).

## Author contributions

J. R. K. S.: conceptualization, data curation, formal analysis, investigation, methodology, validation, visualization, writing – original draft, writing – review & editing. P. S.: conceptualization, formal analysis, investigation, methodology, validation, visualization, writing – review & editing. C. P. C.: methodology, writing – review & editing. M. B. R.: writing – review & editing. M. C. V.: funding acquisition, methodology, writing – review & editing. P. H.: funding acquisition, writing – review & editing. S. G.: conceptualization, funding acquisition, methodology, writing – review & editing. R. K. T.: conceptualization, formal analysis, funding acquisition, methodology, supervision, validation, writing – review & editing.

## Conflicts of interest

The authors declare the following competing interests: S.G. and R.K.T. are founders, shareholders and managing directors of the company 300MICRONS GmbH, active in the field of 3D cell culture platforms.

## Supplementary Material

RA-016-D6RA02741E-s001

## Data Availability

The data underlying this study are available in the published article and its electronic supplementary information (SI). Supplementary information: additional figures and tables (Fig. S1, S2, Tables S1 and S2) presenting photo and height-map of mold, measurement of film thickness and list of analyzed genes and summary of their associated nephron segments and functions. See DOI: https://doi.org/10.1039/d6ra02741e.
