# Supplementary material for: Micro-cold-forming: a simple, rapid, and inexpensive method for the fabrication of microcavities for 3D cell culture
Source: RSC Adv. 2026 May 15;16(28):26068–77. doi: 10.1039/d6ra02741e (PMC13178093; doi:10.1039/d6ra02741e)
Supplement: RA-016-D6RA02741E-s001 [file RA-016-D6RA02741E-s001.pdf]

This journal is © The Royal Society of Chemistry 2026

Micro-cold-forming: A simple, rapid, and inexpensive method for the fabrication of microcavities for 3D cell culture

Jay Rabindra Kumar Samal,[*] Pinak Samal, Carla Pou Casellas, Maarten B. Rookmaaker, Marianne Verhaar, Pamela Habibović, Stefan Giselbrecht and Roman Kurt Truckenmüller[*]

Supplementary information

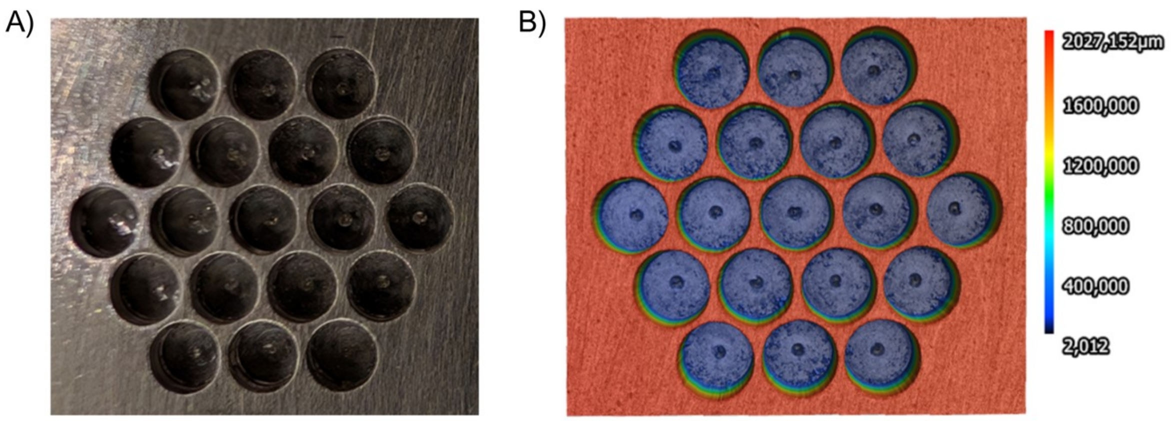

Fig. S1: (A) Photo and (B) optical profilometer-derived height-map of stainless-steel mold used for micro-cold-forming.

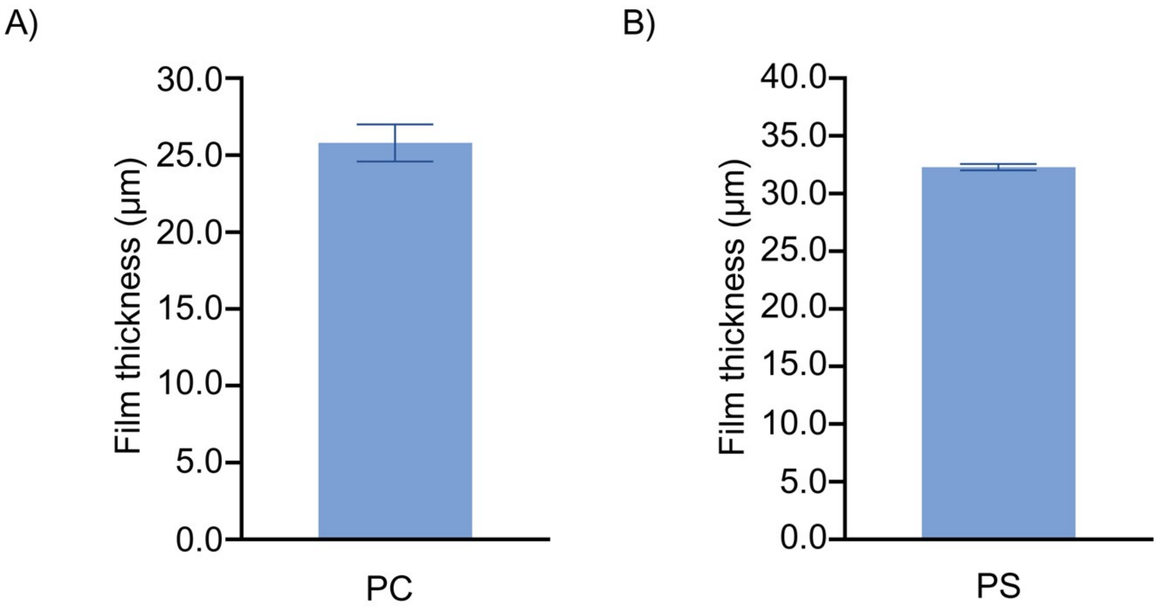

Fig. S2: Measurement of the thickness of the (A) PC and (B) PS film used in this study. Bars and error bars represent mean values ± SD. N = 5.

Table S1: qPCR primers

**Gene**	**Forward primer sequence 5'-3'**	**Reverse primer sequence 5'-3'**
GAPDH	GATTTGGTCGTATTGGGCGC	TTCCCGTTCTCAGCCTTGAC
ANPEP	TGAGCTGTTTGACGCCATCT	GCCCTGCTTGAATACGTCCT
ABCC4	CTCACGCTCATGGGGATGTT	GGTGGTGGGCGTTTCTGATA
SLC4A4	TCTCCAGTGCAAGTAGGATGT	GGTCCTTCTCCGGTTTATCAGA
CLDN10	CCTGGGCTTCTTTGGTTCCA	TCATTGAGCACAGCCCTGAC
PCBD1	GGGTGGAATGAGCTGGAAGG	AGCGTGATGTGGACCTTGTT
AQP3	CTGGATCAAGCTGCCCATCT	CATTGGGGCCCGAAACAAAA
NR3C2	TGTCCTGCTTGCAGACTTCAGA	GCTGCTCCTCGTGAATCCCT
SLC41A3	AGCCGAATCTCAACCTACCTG	CATGGAATTGATTTCTGACGTGC
TRPM7	TCCATTTACACCTGTGCCTCC	GCTCTTCGTAAACCTCCTCCC
HNF1B	ACCAAGCCGGTCTTCCATACT	GGTGTGTCATAGTCGTCGCC
GATA3	GCCCTACTACGGAAACTCGG	GGTGGATGGACGTCTTGGAG
MECOM	TATCCACGAAGAACGGCAATATC	CATGGAAACTTTTGGTGATCTGC

Table S2: Summary of analyzed genes and their associated nephron segments and functions

**Gene**	**Associated nephron segments and functions**
ANPEP	Expressed in proximal tubules and involved in peptide metabolism
ABCC4	Expressed in proximal tubules and collecting ducts and involved in ion transport
SLC4A4	Expressed in proximal tubules and involved in bicarbonate transport and pH regulation
CLDN10	Expressed in thick ascending limb of Henle's loop and involved in tight junction formation and ion permeability
PCBD1	Expressed in distal tubules and involved in transcriptional regulation
AQP3	Expressed in collecting ducts and involved in facilitating water and glycerol transport
NR3C2	Expressed in distal tubules and collecting ducts and important for sodium and water balance
SLC41A3	Expressed in nephron segments and involved in magnesium transport
TRPM7	Expressed in distal tubules and involved in magnesium and calcium transport

HNF1B	Expressed in renal progenitors and epithelial cells and involved in regulation of nephron segment differentiation
GATA3	Expressed in ureteric bud and collecting ducts, involved in branching morphogenesis
MECOM	Expressed in progenitor cells and renal mesenchyme and involved in kidney development
